# Geriatric nutritional risk index and mortality from all-cause, cancer, and non-cancer in US cancer survivors: NHANES 2001–2018

**DOI:** 10.3389/fonc.2024.1399957

**Published:** 2024-06-11

**Authors:** Xiuxiu Qiu, Qidong Wu, Yiyi Zhang, Yingjie Zhu, Ming Yang, Li Tao

**Affiliations:** ^1^ Department of Oncology, Longhua Hospital, Shanghai University of Traditional Chinese Medicine, Shanghai, China; ^2^ Department of Intensive Care Unit, Longhua Hospital, Shanghai University of Traditional Chinese Medicine, Shanghai, China; ^3^ Department of Good Clinical Practice (GCP), Longhua Hospital, Shanghai University of Traditional Chinese Medicine, Shanghai, China

**Keywords:** geriatric nutritional risk index, NHANES, cancer survivors, mortality, older adults

## Abstract

**Background:**

Malnutrition is strongly correlated with worsened treatment outcomes, reduced standard of living, and heightened mortality rates among individuals with cancer. Our research explores how the Geriatric Nutritional Risk Index (GNRI), a measure of nutritional status, relates to all-cause mortality, cancer-specific, and non-cancer mortality among middle-aged and older adult cancer patients.

**Methods:**

We enrolled 3,253 participants aged 40 and above who were diagnosed with cancer. The data was obtained from the National Health and Nutrition Examination Survey (NHANES) dataset covering the period from 2001 to 2018, with a median follow-up duration of 83 months. According to the GNRI levels, patients in the study were classified into two distinct groups: the group with a low GNRI (<98) and the group with a high GNRI (≥ 98). We conducted a Kaplan-Meier survival analysis to assess how survival rates vary with different nutritional conditions. Multivariable Cox regression analyses were performed to estimate hazard ratios (HRs) and 95% confidence intervals (CIs) for all-cause mortality, as well as cancer-specific and non-cancer-related mortality. Restricted cubic spline (RCS) analyses and subgroup evaluations were performed to augment the robustness and validity of our findings.

**Results:**

A total of 1,171 deaths were documented, with 383 attributed to cancer, and 788 from other causes. After adjusting for potential confounders, the analysis demonstrated that, within a specified range, an elevation in the GNRI is inversely associated with mortality from all causes, cancer-specific, and non-cancer causes. Moreover, Kaplan-Meier survival curves for all-cause, cancer-specific, and non-cancer mortality distinctly showed a more pronounced decrease in survival rates among individuals in the low GNRI group (<98). Notably, the restricted cubic spline regression model (RCS) revealed statistically significant non-linear associations between GNRI scores and mortality rates. The *P*-values were ≤0.001 for both all-cause and non-cancer mortality, and 0.024 for cancer-specific mortality.

**Conclusion:**

Our study conclusively demonstrated a robust correlation between GNRI scores and mortality rates among cancer patients, encompassing all-cause mortality as well as specific mortality related to both cancerous and non-cancerous causes. The GNRI may be a valuable prognostic tool for predicting cancer mortality outcomes, offering insights that may inform nutritional management and influence the clinical treatment strategies for cancer survivors.

## Introduction

Cancer continues to be among the most daunting global health challenges, as recent years have shown an alarming increase in mortality rates. According to the World Health Organization (WHO) projections, the number of new cancer cases reported yearly is expected to rise to 28.4 million by 2040 (1). It is estimated that by 2024, 611,720 people will die from cancer in the United States, which equates to about 1,680 deaths per day (2). This increasing trend in cancer mortality not only underscores the critical importance of ongoing research into cancer causes, prevention, and treatment but also highlights the necessity for public health initiatives aimed at reducing risk factors among populations.

Cancer survivors often encounter various challenges after treatment, including physical, psychological, and social obstacles. Maintaining optimal nutritional status is a crucial aspect of survivorship care. Malnutrition becomes increasingly prevalent among both older individuals and cancer patients as cancer progresses. Previous studies have shown that malnutrition in cancer patients can lead to increased postoperative complications, prolonged hospitalization, poor treatment outcomes, and higher mortality rates (3). Research suggests that the occurrence of malnutrition among individuals with cancer shows significant variation, with rates ranging from 20% to 70% (4). A study spanning all regions of Brazil revealed that 45.3% of patients with cancer who are admitted to the hospital, when evaluated utilizing the Patient-Generated Subjective Global Assessment (PG-SGA), exhibited varying levels of malnutrition, including moderate and severe categories (5). Recently, Geriatric nutritional risk index(GNRI) has gained widespread recognition for its significant correlation with mortality rates in older cancer patients across multiple cancer types (6).

First introduced in 2005, the Geriatric Nutritional Risk Index (GNRI) is a scientifically proven tool created to evaluate the nutritional condition of older adult populations (7). The GNRI utilizes two critical parameters for assessment: serum albumin levels and body weight. The instrument has been essential in predicting patient outcomes for various types of cancers, such as gastric cancer (6), hepatocellular carcinoma (8), and head and neck cancer (9), underscoring its importance. Additionally, it has been associated with medical outcomes that occur after surgical procedures, chemotherapy treatments, or a combination of chemotherapy and radiation therapy for many types of malignancies (10).

Given these considerations, this study assessed the relationship between GNRI and mortality attributable to all causes, cancer-related and non-cancer causes, within a sample of middle-aged and older adult cancer patients in the United States that accurately represents the entire population. The objective is to offer innovative perspectives on nutritional and supportive care strategies for individuals with cancer.

## Materials and methods

### Data sources

The National Health and Nutrition Examination Survey (NHANES) database, accessible at (https://www.cdc.gov/nchs/nhanes/index.htm), provides extensive coverage through various indicators. It enables the retrieval of detailed demographic statistics, comprehensive socioeconomic information, dietary and health data, physiological metrics, laboratory test results, and other related data, encompassing a broad cross-section of the United States. Administered by the Centers for Disease Control and Prevention (CDC) and the National Center for Health Statistics (NCHS), NHANES aims to provide comprehensive, nationally representative data on the health and nutritional status of the civilian population in the United States. Before participating, all subjects provided informed consent, and the NCHS Ethics Review Board approved the data collection methodologies used in NHANES.

### Study design and population

**Figure 1:**
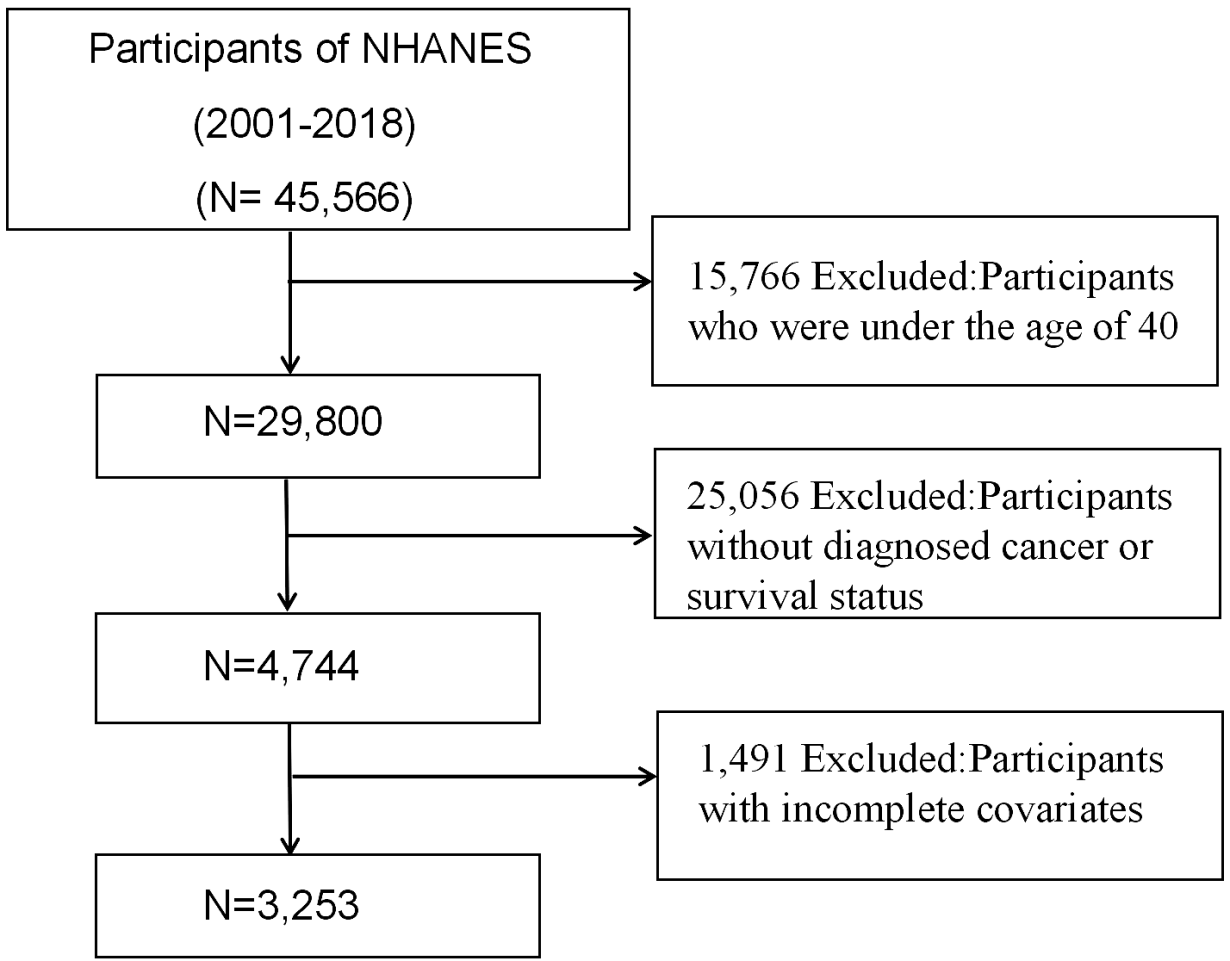
Flowchart of the participant’s selection from NHANES 2001-2018.

This investigation included 45,566 individuals who participated in nine consecutive NHANES survey cycles from 2001 to 2018. Individuals under 40 (n=15,766) and those without a cancer diagnosis or known survival status (n=25,056) were excluded from the study. Additionally, we excluded participants lacking GNRI-related data or those with incomplete covariates (n=1,491). After applying the above criteria, 3,253 cancer survivors were available for analysis. **Figure 1** depicts a flowchart illustrating the criteria employed for patient selection.

### Definition of geriatric nutritional risk index

The GNRI (Geriatric Nutritional Risk Index) is a validated, straightforward scoring system developed specifically to assess the nutritional status of patients across various medical settings, notably among those undergoing surgical procedures. This index is highly precise, relying solely on objective parameters such as serum albumin levels, height, and weight, which are readily available from standard laboratory tests. The formula used to calculate the GNRI is: GNRI = (1.489 × serum albumin level in g/L) + (41.7 × actual body weight/ideal body weight in kg), where a ratio of actual to ideal weight set at 1 is assumed. This method has been recognized as a significant predictor of outcomes in diverse cancer populations, illustrating its broad applicability and reliability (11). For practical application, GNRI scores lead to the classification of patients into two primary groups: those with a GNRI < 98 form the Low-GNRI group indicating higher nutritional risk, and those with a GNRI ≥ 98 compose the High-GNRI group suggesting lower nutritional risk (12–14). To refine the understanding of nutritional risk gradients, GNRI scores were further segmented into three tertiles. This categorization helps clinicians identify patients at varying degrees of malnutrition risk more distinctly, with Tertile 1 representing the highest risk and Tertile 3 the lowest, thus enabling more targeted nutritional interventions based on the severity of risk (14).

### Ascertainment of death

The mortality data for this analysis were obtained from the NHANES Public Use Linked Mortality File and integrated with standard NHANES datasets using the unique respondent sequence number assigned to each participant. The analysis encompassed mortality status and follow-up duration, classifying outcomes into two categories: survival and death. Cancer mortality is defined as the likelihood of dying from various malignant tumors, whereas non-cancer mortality refers to the likelihood of death from causes other than cancer. The National Death Index (NDI) records were used to determine the mortality status and causes of death until 31 December 2019. In the 10th edition of the International Classification of Diseases (ICD-10), it is outlined that deaths attributed to malignant neoplasms are categorized as cancer mortality (with the specific codes of ICD-10 C00-C97) (15).

### Cancer patients

Patients with a previous diagnosis of cancer were identified through the inquiry: “Have you ever been told by a doctor that you had cancer or a malignancy of any kind?” Individuals responding “YES” were selected for inclusion. Following this selection, cancer patients were further queried regarding the specific type of cancer diagnosed.

### Covariates

Data regarding the demographic characteristics of the patients, including age, gender, race, education level, and marital status, was collected. Additional covariates were derived from physical examinations conducted at a mobile examination center and from laboratory test results. Ethnicity was divided into four distinct groups: Mexican American, Non-Hispanic White, Non-Hispanic Black, and Other. Education levels were dichotomized as “Below High School” and “High School or Above.” Marital status was categorized into four groups: Married/Cohabitant, Widowed, Separated/Divorced, and Never Married. Body Mass Index (BMI) was determined by dividing weight in kilograms (kg) by the square of height in meters (m²). Based on these calculations, BMI values were categorized into three groups: Underweight or Normal (<25.0 kg/m²), Overweight (25.0 to 30.0 kg/m^2^), and Obesity (≥30.0kg/m²) (16). Physical activity was classified as either moderate/vigorous recreational activities (yes) or none. Additionally, the energy intake was assessed using dietary data obtained from 24-hour dietary recalls. Smoking status was classified into three categories: Never Smoker, Former Smoker, and Current Smoker. A “Never Smoker” is defined as an individual who has smoked fewer than 100 cigarettes in their lifetime. A “Former Smoker” refers to someone who has smoked more than 100 cigarettes in their lifetime but has quit smoking. A “Current Smoker” is defined as someone who has smoked more than 100 cigarettes in their lifetime and continues to smoke occasionally or daily. Alcohol consumption was categorized based on the past year’s responses into five groups: Never (fewer than 12 drinks in a lifetime), Mild (≤ 2 drinks every day for males, ≤ 1 drink every day for females), Moderate (≤ 3 and > 2 drinks every day for males, ≤ 2 and > 1 drink every day for females, or ≥ 2 to < 5 days per month of binge drinking), Heavy (≥ 4 drinks every day for males, ≥ 3 drinks every day for females, or ≥ 5 days per month of binge drinking), and Former (no drinking in the past year but over 12 drinks in any previous year). The study identified chronic diseases, including diabetes, hypertension, coronary heart disease (CHD), and stroke. Diabetes was further classified into diabetes mellitus (DM), compromised fasting glycemia, and impaired glucose tolerance. Diagnosis of these conditions relied on patient self-reporting.

### Statistical analysis

In this study, we rigorously adhered to the NHANES complex survey design principles, taking into account weights, clustering, and stratification to ensure representative and accurate analyses. We initiated our analysis by categorizing cancer survivors based on their Geriatric Nutritional Risk Index (GNRI) scores into two groups: Low GNRI (< 98) and High GNRI (≥98). For the descriptive analysis, we employed the weighted mean ± standard deviation to summarize continuous variables, ensuring that the complex survey design was accounted for in these estimations. Categorical variables were reported as frequencies and percentages, with survey design adjustments applied to accurately reflect the NHANES sample population. This method enabled a comprehensive comparison of baseline characteristics between the Low GNRI and High GNRI groups, utilizing χ2 tests for categorical variables and ANOVA for continuous variables.

Next, in the analysis of the relationship between GNRI and mortality risk among cancer patients, Cox proportional hazards regression models were employed. The Cox regression model was used to estimate hazard ratios (HRs) and 95% confidence intervals (CIs). The proportional hazards assumption was tested using the Schoenfeld residuals method. These models facilitated the examination of GNRI both as a continuous variable, to assess the impact of each unit change in GNRI on mortality risk, and as a categorical variable, to discern potential nonlinear relationships and risk distribution. Model 1 incorporated age, ethnicity, and gender as variables and accounted for them through modifications. Model 2 expanded upon the changes made in Model 1 by including additional factors such as BMI, marital status, education level, drinking status, smoking behaviors, and the Poverty Income Ratio (PIR). Model 3 extended the range of adjustments to encompass energy intake, physical activity, hypertension, coronary heart disease (CHD), diabetes, stroke, and tumor types, in addition to those already considered in Model 2. This approach allowed us to reveal subtle trends and potential risk thresholds, while thoroughly accounting for a wide spectrum of potential confounders that could influence this relationship.

Kaplan-Meier curves were generated to illustrate the survival probabilities for the three groups based on GNRI status, depicting all-cause mortality, cancer-specific mortality, and non-cancer mortality. Survival distributions across these categories were compared using the log-rank test. Additionally, a restricted cubic spline (RCS) regression with four knots was conducted to assess the non-linear relationships between GNRI and mortality risk. Upon detecting nonlinearity, two-piecewise Cox proportional hazards regression models were constructed, centered around the inflection point. In the subgroup analysis, we further explored the relationship between GNRI scores and mortality risk across various demographic and clinical subgroups. This process entailed stratifying the sample based on factors such as age, gender, ethnicity, and comorbid conditions, and evaluating the association between GNRI scores and mortality within each stratum. We utilized interaction tests to determine if the effect of GNRI on mortality varied significantly across these subgroups, as presented in forest plots. The *P*-interaction values were derived from Cox proportional hazards regression models, which were adjusted for the complex survey design to ensure that our findings accurately reflected the diversity of the NHANES population. Statistical analyses were performed with version 4.3.2 of R software (R Foundation for Statistical Computing). Weighted regression analysis utilized the “survey” package; construction of the RCS regression harnessed the “rms” package. In this investigation, a two-sided *P*-value of <0.05 was considered to be statistically significant.

## Results

### Baseline characteristics of study participants

A total of 3,253 cancer survivors were included in this study, representing a weighted population of 15,723,705 individuals. The weighted mean (standard error) age was 64.78 (0.30) years, with 44.1% male and 55.9% female. **Table 1** presents a comparison of the characteristics between the High-GNRI group (GNRI ≥ 98, n = 2963) and the Low-GNRI group (GNRI < 98, n = 290). The majority of patients were non-Hispanic White (88.38%). The *p*-values indicated significant differences in ethnicity, education level, PIR, BMI, smoking status, diabetes, and tumor types among the groups (*P* < 0.05). However, age, gender, marital status, drinking status, energy intake, physical activity, hypertension, stroke, and CHD did not exhibit significant differences between the two groups concerning the nutritional risk indicated by the GNRI score (*P* > 0.05). Significantly, the high GNRI group (GNRI ≥ 98) primarily comprised non-Hispanic White individuals who possessed at least a high school education and consumed more energy. These individuals were either former or never smokers, were identified as mild drinkers, had a higher BMI, and did not have diabetes. Additionally, a higher proportion of participants with gastrointestinal (5.94%) skin (38.48%) and gynecologic (37.05%) cancers were included.

**Table 1:** Baseline characteristics of cancer patients ≥40 y by GNRI groups in NHANES 2001–2018.

Characteristics	Total	GNRI Groups		*P-*value
		Low-GNRI (GNRI < 98)	High-GNRI (GNRI≥98)	
**Number**	3253	N=290	N=2963	
**Age,mean**	64.78 ± 0.30	65.60 ± 1.07	64.69 ± 0.30	0.397
<60	761(33.74)	61(31.50)	700(33.91)	0.564
≥60	2492(66.26)	229(68.50)	2263(66.09)	
**Gender,%**				0.846
Male	1610(44.13)	159(44.87)	1451(44.07)	
Female	1643(55.87)	131(55.13)	1512(55.93)	
**Ethnicity,%**				**<0.001**
Mexican American	183(1.67)	11(2.11)	172(1.64)	
Non-Hispanic Black	449(5.07)	75(11.82)	374(4.56)	
Non-Hispanic White	2343(88.38)	172(79.23)	2171(89.07)	
Other	278(4.87)	32(6.83)	246(4.72)	
**Education level,%**				0.039
High school or above	2949(95.32)	256(92.90)	2693(95.50)	
Below high school	304(4.68)	34(7.10)	270(4.50)	
**PIR**	3.36 ± 0.04	2.98 ± 0.12	3.39 ± 0.04	0.002
**BMI**				**< 0.001**
Underweight or normal	905(28.19)	132(43.54)	773(27.03)	
Over weight	1182(35.35)	86(27.66)	1096(35.53)	
Obese	1166(36.47)	72(28.80)	1094(37.05)	
**Martial status,%**				0.526
Married or cohabitant	2009(66.69)	161(63.77)	1848(66.91)	
Divorced or separated	512(14.79)	59(17.04)	453(14.62)	
Widowed	585(14.47)	58(16.00)	527(14.36)	
Never married	147(4.05)	12(3.19)	135(4.12)	
**Drinking status,%**				0.058
Former	833(21.07)	100(29.07)	733(20.47)	
Heavy	247(8.62)	23(8.28)	224(8.65)	
Mild	1395(46.03)	105(41.90)	1290(46.34)	
Moderate	353(14.20)	22(8.99)	331(14.60)	
Never	425(10.08)	40(11.76)	385(9.95)	
**Smoking status,%**				0.012
Former	1407(41.22)	133(45.86)	1274(40.87)	
Never	1407(44.50)	101(34.04)	1306(45.29)	
Now	439(14.28)	56(20.10)	383(13.84)	
**Energy intake,%**	1953.66 ± 21.13	1864.23 ± 62.85	1960.42 ± 21.67	0.136
**Physical activity,%**				0.670
No	2697(82.39)	251(83.71)	2446(82.29)	
Yes	556(17.61)	39(16.29)	517(17.71)	
**Hypertension,%**				0.424
No	1070(38.59)	96(35.76)	974(38.80)	
Yes	2183(61.41)	194(64.24)	1989(61.20)	
**Diabetes,%**				**< 0.001**
DM	877(22.84)	67(19.58)	810(23.09)	
IFG	179(5.66)	2(0.53)	177(6.05)	
IGT	138(3.71)	8(1.93)	130(3.84)	
No	2059(67.79)	213(77.96)	1846(67.02)	
**Stroke,%**				0.565
No	2965(93.51)	264(92.61)	2701(93.59)	
Yes	287(6.48)	25(7.39)	262(6.41)	
**CHD,%**				0.752
No	2911(93.51)	259(90.96)	2652(91.57)	
Yes	342(6.48)	31(9.04)	311(8.43)	
**Type of cancer,%**				**< 0.001**
Gastrointestinal	266(5.94)	36(10.94)	230(5.56)	
Gynecologic	1377(37.05)	131(45.81)	1246(36.38)	
Head and neck	97(3.26)	9(2.53)	88(3.31)	
Hematologic	102(3.22)	17(6.34)	85(2.98)	
Respiratory system	84(2.18)	20(7.15)	64(1.80)	
Skin cancers	990(38.48)	40(15.92)	950(40.19)	
Urologic tumors	98(3.04)	9(4.59)	89(2.92)	
Other cancers	239(6.85)	28(6.72)	211(6.86)	

Continuous variables were expressed as weighted mean ± standard deviation; Categorical variables were expressed as weighted frequencies and percentages. Bold values indicate statistical significance (P < 0.05).

GNRI, geriatric nutritional risk index; BMI, Body Mass Index; CHD, Coronary Heart Disease; PIR, the ratio of family income to poverty; IFG, impaired fasting glucose; IGT, impaired glucose tolerance; DM: diabetes mellitus.

### Survival analysis of the GNRI in assessing mortality risk

**Figure 2:**
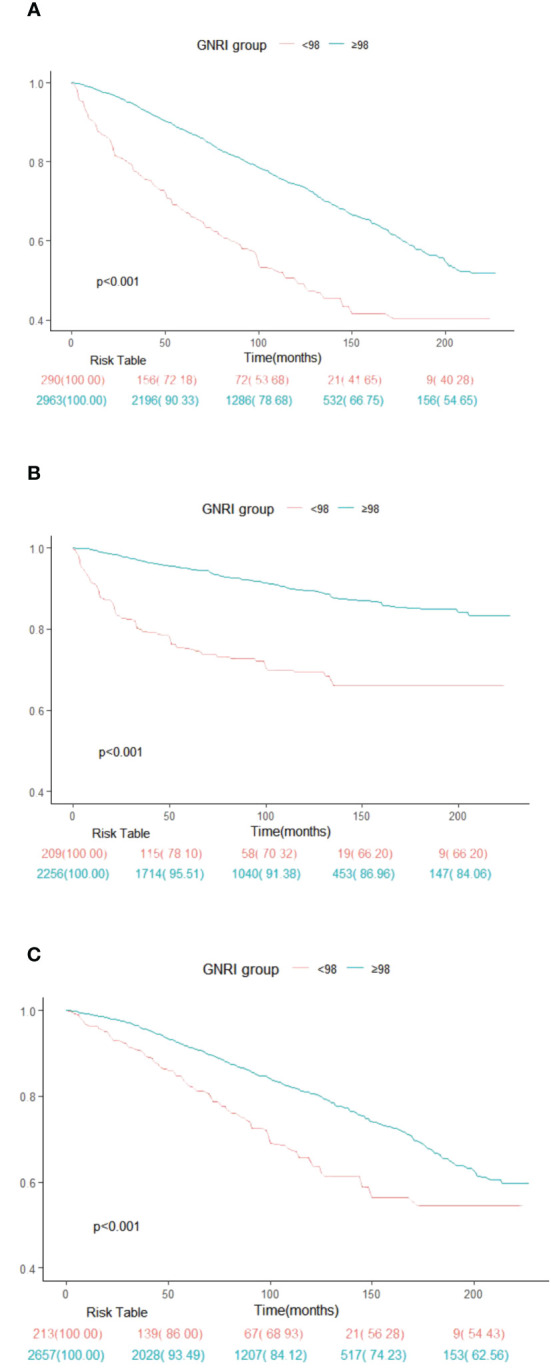
Kaplan-Meier survival rates illustrating mortality among US cancer patients categorized by different Geriatric nutritional risk index (GNRI) groups: **(A)** all cause mortality; **(B)** cancer mortality; **(C)** non-cancer mortality. In the Kaplan-Meier curves, the population is stratified into twor groups (GNRI <98, and GNRI ≥98), and statistical analysis is conducted using the long-rank test.

**Figure 2** depicts Kaplan-Meier survival curves for all-cause, cancer, and non-cancer mortality of survival among American adult cancer patients stratified by the GNRI group (**Figures 2A–C**). The survival analysis elucidated significant differences in survival probabilities between these cohorts. Notably, the cohort with GNRI scores < 98 exhibited substantially reduced survival rates across all evaluated mortality domains, compared to their counterparts in the ≥98 GNRI cohort. This discrepancy in survival outcomes was statistically significant, as evidenced by the log-rank test results, which yielded p-values of <0.001 for all-cause, cancer, and non-cancer mortality.

### Association between GNRI and mortality

After adjusting for multiple covariates, a multivariate analysis was conducted using three distinct regression models to investigate the predictive value of GNRI on overall mortality, cancer-related mortality, and non-cancer-related mortality. The GNRI is assessed both as a continuous variable and in categorized forms. The findings are displayed as hazard ratios (HRs) alongside their corresponding 95% confidence intervals (CIs) (**Table 2**).

**Table 2:** COX proportional hazard regression analysis of GNRI and all-cause mortality, cancer and non-cancer mortality in patients with cancer.

Variables	Model 1		Model 2		Model 3	
All-cause mortality	HR(95%CI)	*P*	HR(95%CI)	*P*	HR(95%CI)	*P*
GNRI	0.99(0.98,0.99)	<0.001	0.99(0.98,0.99)	<0.001	0.99(0.98,0.99)	<0.001
GNRI (Category)						
GNRI< 98	Reference		Reference		Reference	
GNRI≥ 98	0.43(0.35,0.54)	<0.001	0.49(0.39,0.63)	<0.001	0.49(0.38,0.62)	<0.001
GNRI Classification						
Tertile1[33.70,112.97]	Reference	–	Reference	–	Reference	–
Tertile2(112.97,123.12]	0.61(0.52,0.72)	<0.001	0.62(0.51,0.76)	<0.001	0.61(0.50,0.75)	<0.001
Tertile3(123.12,203.79]	0.56(0.47,0.66)	<0.001	0.50(0.38,0.65)	<0.001	0.51(0.39,0.66)	<0.001
*p* for trend	<0.001		<0.001		<0.001	
Cancer mortality	HR(95%CI)	*P*	HR(95%CI)	*P*	HR(95%CI)	*P*
GNRI	0.98(0.98,0.99)	<0.001	0.98(0.98,0.99)	<0.001	0.98(0.98,0.99)	<0.001
GNRI (Category)						
GNRI< 98	Reference		Reference		Reference	
GNRI≥ 98	0.27(0.19,0.38)	<0.001	0.32(0.22,0.47)	<0.001	0.34(0.23,0.50)	<0.001
GNRI Classification						
Tertile1[33.70,112.97]	Reference	–	Reference	–	Reference	–
Tertile2(112.97,123.12]	0.46(0.35,060)	<0.001	0.45(0.34,0.61)	<0.001	0.44(0.32,0.61)	<0.001
Tertile3(123.12,203.79]	0.46(0.35,0.61)	<0.001	0.35(0.22,0.55)	<0.001	0.37(0.23,0.59)	<0.001
*p* for trend	<0.001		<0.001		<0.001	
Non-cancer mortality	HR(95%CI)	*P*	HR(95%CI)	*P*	HR(95%CI)	*P*
GNRI	0.99(0.99,1.00)	<0.001	0.99(0.99,1.00)	0.010	0.99(0.99,1.00)	0.010
GNRI (Category)						
GNRI< 98	Reference		Reference		Reference	
GNRI≥ 98	0.56(0.44,0.73)	<0.001	0.63(0.48,0.82)	<0.001	0.60(0.45,0.79)	0.001
GNRI Classification						
Tertile1[33.70,112.97]	Reference	–	Reference	–	Reference	–
Tertile2(112.97,123.12]	0.66(0.53,0.82)	<0.001	0.67(0.52,0.85)	<0.001	0.66(0.52,0.82)	<0.001
Tertile3(123.12,203.79]	0.56(0.45,0.71)	<0.001	0.54(0.39,0.76)	<0.001	0.53(0.38,0.75)	<0.001
*p* for trend	<0.001		<0.001		<0.001	

Calculated using multivariate COX regression analysis was performed. Model 1: adjusted for age, gender, and ethnicity; Model 2: Additionally adjusted for Body Mass Index(BMI), marital status, education level, drinking status, smoking status, and the ratio of family income to poverty(PIR) based on Model 1. Model 3: Additionally adjusted for energy intake, physical activity, hypertension, diabetes, stroke, and coronary. heart. Disease (CHD) and tumor types based on Model 2.

Across all models, a higher GNRI, treated as a continuous variable, was associated with a statistically significant reduction in the risk of all-cause mortality (HR < 1, *P* < 0.001), cancer mortality (HR < 1, *P* < 0.001), and non-cancer mortality (HR < 1, *P* < 0.001). This indicates that higher GNRI scores are protective against mortality. In categorical analyses, individuals with a GNRI ≥98 had significantly lower hazard ratios (HRs) for all-cause, cancer-specific, and non-cancer mortality compared to the reference group (GNRI < 98), with HRs of 0.49(95% CI: 0.38,0.62),0.34(95% CI: 0.23,0.50), and 0.60(95% CI: 0.45,0.79) in Model 3, respectively.

The stratification of GNRI resulted in the identification of three distinct categories: Tertile 1 (33.70, 112.97], Tertile 2 (112.97, 123.12], and Tertile 3 (123.12, 203.79]. Tertile 1 served as the referential baseline. After adjusting for potential confounding variables, observations across the three models unveiled a consistent trend. Specifically, in the most adjusted Model 3, the HRs for all-cause, cancer-specific, and non-cancer mortality among individuals in Tertile 3, compared with Tertile 1, were 0.51 (95% CI: 0.39–0.66), 0.37 (95% CI: 0.23–0.59), and 0.53 (95% CI: 0.38–0.75), respectively. The significance of this trend was supported by *P*-values for the trend, which were <0.001 for all mortality outcomes in every model, thereby strengthening the correlation between GNRI scores and risk of mortality.

### Nonlinear relationship between GNRI and mortality risk

**Figure 3:**
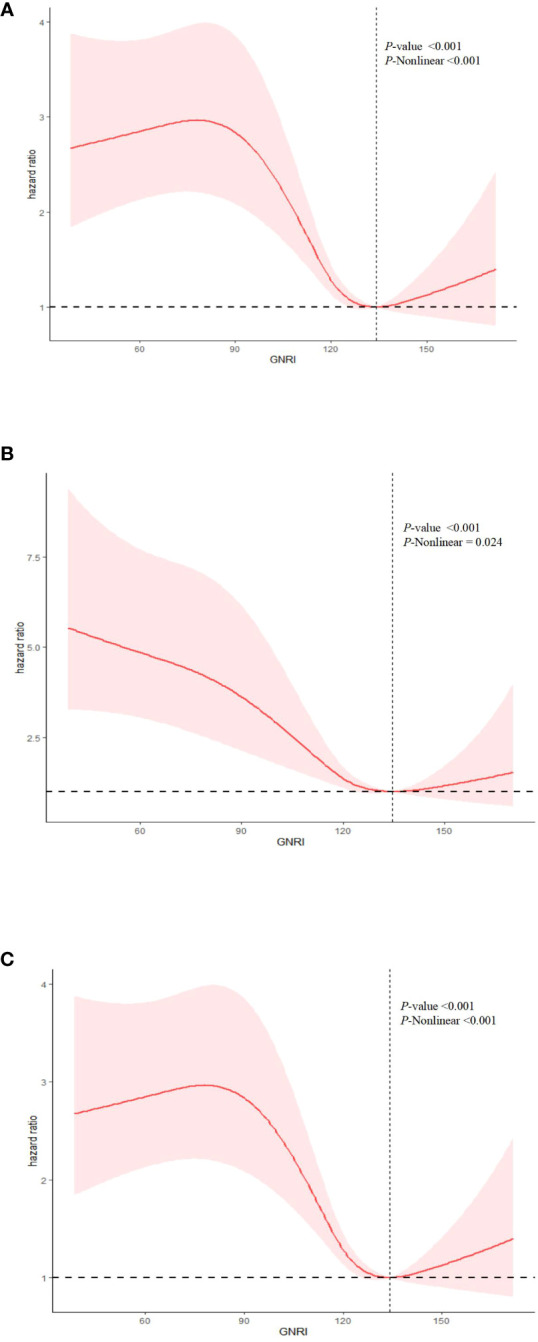
Nonlinear relationship between GNRI and mortality in cancer patients. **(A)** all cause mortality; **(B)** cancer mortality; **(C)** non-cancer mortality. Statistical adjustments were made for age, gender, marital status, ethnicity, education level, Body Mass Index (BMI), smoking status, drinking status, energy intake, physical activity, the ratio of family income to poverty (PIR), diabetes, hypertension, coronary heart disease (CHD), stroke, and tumor types.

In a fully adjusted restricted cubic spline regression model (RCS), we detected a significant nonlinear relationship between GNRI and all-cause, cancer-specific, and non-cancer mortality among cancer patients. This was evidenced by the *P*-Nonlinear values of <0.001 for all-cause mortality, 0.024 for cancer-specific mortality, and <0.001 for non-cancer mortality (**Figure 3**). The RCS analysis indicates that patients with extremely low or high GNRI scores are significantly associated with an increased risk of mortality for all-cause, cancer-specific, and non-cancer mortality. It also shows that as the GNRI increases to a certain point, mortality risk decreases before slightly increasing again. This suggests that maintaining a GNRI score around this optimal value may benefit patients, scores outside this range are associated with a higher mortality risk.

After identifying a non-linear relationship, we utilized two segmented linear regression models to delineate the changes in the relationship between GNRI and mortality risk at specific threshold points (**Table 3**). Thresholds were determined by evaluating all possible values and selecting the points with the highest log-likelihood values. The established thresholds were 134.16 for all-cause mortality, 134.65 for cancer mortality, and 134.15 for non-cancer mortality, indicating significant changes in the linear relationship between GNRI and mortality risk. For all-cause mortality, when GNRI was below the threshold of 134.16, each unit increase resulted in a slight but statistically significant reduction in mortality risk, with a hazard ratio (HR) of 0.99 (95% CI: 0.98, 0.99). Above this threshold, the HR was 1.00 (95% CI: 0.98, 1.02), suggesting that further increases in GNRI did not significantly alter mortality risk. In the models for cancer mortality and non-cancer mortality, the HR below the thresholds also indicated a statistically significant risk reduction.

**Table 3:** Nonlinearity addressed through a two-piecewise linear model.

	All-cause mortality	*P*-value	Cancer mortality	*P*-value	Non-cancer mortality	*P*-value
Threshold value	134.16		134.65		134.15	
<Threshold value	0.99(0.98, 0.99)	<0.001	0.99(0.98, 0.99)	<0.001	0.99(0.98,0.99)	<0.001
>Threshold value	1.00(0.98, 1.02)	0.960	1.02(0.98, 1.03)	0.750	1.00(0.98,1.02)	0.960
*P* for log likehood Ratio test	<0.001		0.024		<0.001	

Hazard ratios were calculated using a multivariable Cox proportional hazards regression model, adjusted for age, gender, marital status, education level, ethnicity, drinking status, smoking status, family income-to-poverty ratio (PIR), Body Mass Index (BMI), energy intake, physical activity, hypertension, diabetes, stroke, coronary heart disease (CHD), and tumor types.

The *P*-values for the log-likelihood ratio tests were statistically significant across all outcomes (all-cause and non-cancer mortality <0.001, cancer mortality 0.024), confirming that the segmented linear models were more suitable for these data than the basic linear models and that there is indeed a non-linear association between GNRI and various forms of mortality.

### Subgroup analysis

#### Stratified analysis of the association between GNRI and mortality outcomes

**Figure 4:**
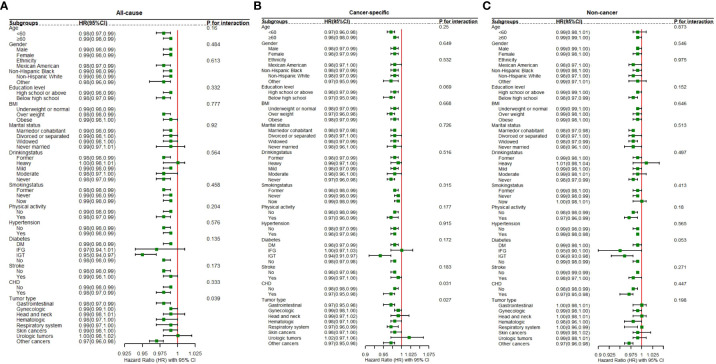
The figure show the association between GNRI and risk of mortality in different participants for **(A)** all cause mortality; **(B)** cancer-specific mortality; and **(C)** non-cancer mortality. The analysis was adjusted for age, gender, marital status, education level, ethnicity, drinking status, smoking status, Body Mass Index (BMI), and physical activity, hypertension, diabetes, stroke, coronary heart disease (CHD), and tumor types.

**Figure 4** elucidates the results from subgroup analyses investigating the association between GNRI and three categories of mortality: **Figure 4A**. all-cause, **Figure 4B**. cancer-related, and **Figure 4C**. non-cancer-related mortality. These analyses were stratified by several key variables, including age, gender, marital status, educational level, BMI, smoking status, drinking status, and medical history (stroke, hypertension, diabetes, coronary heart disease). Additionally, the analysis considered tumor type in the context of cancer mortality. The figures display hazard ratios (HRs) along with 95% confidence intervals (CIs) to quantify the strength and direction of associations within each subgroup. *P*-values for interaction are used to assess the statistical significance of differences in the GNRI-mortality association across subgroups.

Significant interactions were observed between GNRI and specific factors. Notably, the association between GNRI and all-cause mortality varied significantly across different tumor types (*P* for interaction < 0.05), underscoring the impact of cancer pathology on nutritional risk and survival outcomes. Similarly, a significant interaction was identified between cancer mortality and coronary heart disease (CHD) (*P* for interaction < 0.05), suggesting that cardiovascular comorbidities may modulate the impact of nutritional status on cancer survival. Conversely, no significant interactions were detected between GNRI and other examined factors—age, gender, marital status, education level, BMI, smoking status, drinking status, physical activity, and medical history (stroke, hypertension and diabetes)—for all-cause, cancer-specific, and non-cancer mortality (*P* for interaction > 0.05). This indicates that the effect of GNRI is generally consistent across these demographic and clinical characteristics, underscoring its broad applicability as a prognostic tool in diverse patient populations. The findings from these subgroup analyses are crucial for identifying populations at heightened risk and for tailoring interventions that enhance nutritional status and, consequently, improve patient outcomes.

## Discussion

In this comprehensive retrospective cohort study, we elucidated the non-linear relationship between GNRI and mortality risk among cancer survivors. This included all-cause, cancer-specific, and non-cancer mortality, identifying an optimal GNRI range associated with the lowest risk of death for patients. In the analysis of continuous variables, we observed that each additional unit of GNRI is associated with a reduction in mortality risk. However, this relationship reverses when GNRI values are extremely low or high, as demonstrated through the analysis of categorical variables. Analyzing GNRI as a continuous variable provides a comprehensive perspective, helping to reveal the overall risk curve, while categorical analysis highlights specific risk intervals. Using both models together offers the greatest insights and guidance for clinical practice. Additionally, it is worth noting that subgroup analyses have confirmed GNRI’s predictive consistency across different patient demographics. These findings indicate that GNRI serves as an independent predictor of mortality in cancer survivors. Therefore, a heightened focus should be placed on assessing the nutritional status using GNRI to mitigate mortality within the cancer population (17).

The global cancer burden continues to rise, with significant variability in cancer incidence and mortality rates between regions and countries. Malnutrition is notably prevalent among tumor patients, and nutritional status is acknowledged as an essential factor that determines the level of well-being and life satisfaction in these individuals (18). Comprehensive nutritional assessments and interventions are necessary.

The Geriatric Nutritional Risk Index (GNRI) is distinguished from other inflammatory indices and nutritional indicators by its several significant advantages. Firstly, the GNRI is a simple and accurate tool that requires only routine measurements of albumin and Body Mass Index (BMI) (19). In contrast, other indices such as C-reactive protein (CRP) or the Nutritional Risk Index (NRI), often necessitate more complex or less accessible data. Secondly, recent studies have highlighted the value of GNRI in assessing the prognosis of cancer patients (20). It includes not only an assessment of nutritional status but also takes into account age factors, making it more targeted in prognostic predictions for elderly cancer patients. Ruan et al. found that the GNRI is a key prognostic indicator for survival in elderly cancer patients with cachexia (17). Nakayama et al. found that a low GNRI is significantly associated with a higher mortality risk in patients with advanced head and neck cancer (9). Additionally, GNRI has been identified as a significant predictor of postoperative respiratory complications in esophageal cancer patients and overall survival in lung cancer patients (21, 22). This is in contrast to other commonly used nutritional indices, such as the Prognostic Nutritional Index (PNI) and the Controlling Nutritional Status (CONUT) score. Wang et al. (23) determined that, according to the ESPEN 2015 guidelines, the GNRI is the most accurate tool for predicting malnutrition in esophageal cancer patients. The GNRI combines nutritional and inflammatory indicators by including serum albumin, which reflects both nutrition and inflammation. This broader approach renders the GNRI more comprehensive than indices such as the Neutrophil-to-Lymphocyte Ratio (NLR) or C-reactive protein (CRP), which mainly assess inflammation and neglect nutritional status.

Therefore, nutritional status may serve as a predictor of the efficacy of cancer therapy (24). The GNRI may be an effective marker for evaluating nutritional risk in cancer patients, with lower scores being correlated with higher rates of treatment complications, poorer responses to therapy, and decreased survival rates. Moreover, numerous studies have aimed at improving outcomes for malnourished patients, and comprehensive nutritional support guidelines have been published. Tumor-related inflammation and malnutrition are strongly linked to tumor recurrence and progression in cancer patients (25). Further analysis of how GNRI predicts cancer prognosis suggests that malnutrition is linked to a lower quality of life, less successful treatment outcomes, and an increased likelihood of mortality (26). We argue that GNRI can serve as a critical tool in assessing the prognosis of individual cancer patients. It is critically important to emphasize the nutritional status of middle-aged and older adult cancer patients.

This study establishes a link connection between GNRI levels and mortality among individuals with cancer. By utilizing nationally representative sample data from the NHANES database, our findings have the potential to impact the entire cancer survivor population in the country. This provides nutrition recommendations with significant reference value, underscoring the importance of work involving cancer survivors. For example, patients with extremely low GNRI should be given priority for nutritional support to raise their GNRI to an optimal range. Conversely, for patients with extremely high GNRI, it may be necessary to evaluate for overnutrition or other risk factors. Our analysis emphasizes that moderately adjusting GNRI to a relatively safe range could be a crucial strategy for improving the survival quality and prognosis of older adult patients.

However, it is important to acknowledge certain limitations. Tumors exhibit varied degrees of malignancy, and patients undergo a range of treatments. Additionally, the GNRI assessment was conducted only once, without monitoring changes over time during the follow-up period. GNRI relies solely on serum albumin levels and BMI, making it impossible to account for total body composition, including muscle mass, fat, and total body water. Despite adjustments for many well-known confounders, certain unknown factors could not be accounted for. Furthermore, using GNRI as the sole measure of nutritional status fails to capture comprehensive aspects of nutritional health, such as micronutrient status and dietary intake patterns. Therefore, conducting further prospective randomized controlled studies to validate our findings is crucial, emphasizing the urgency and importance of future research in this area.

## Conclusion

Based on existing evidence, our findings revealed a strong association between GNRI levels and the risks of all-cause, cancer-specific, and non-cancer mortality among cancer survivors. However, it is crucial to note that this association appears to exhibit a nonlinear characteristic at very low (GNRI < 90) or very high (GNRI > 130) values, where individuals with GNRI values in these extreme ranges may face an increased risk of mortality. The clinical significance of this finding is that it advises physicians and nutrition experts to strive to maintain patients’ GNRI within an optimal range, rather than merely seeking to increase or decrease the GNRI unidirectionally. Understanding the association between GNRI and mortality among cancer survivors could aid in stratifying patients based on their risk, leading to more personalized survivorship care plans. Our findings offer new perspectives on dietary and nutritional approaches for those who have survived cancer, potentially informing personalized interventions that could improve outcomes and quality of life for this population.

## Data availability statement

The original contributions presented in the study are included in the article/supplementary material. Further inquiries can be directed to the corresponding author/s.

## Ethics statement

The National Center for Health Statistics Research Ethics Review Board reviewed and approved the studies involving human participants. All participants provided their written informed consent to participate in this study.

## Author contributions

XQ: Writing – original draft, Methodology, Writing – review & editing, Resources. QW: Data curation, Writing – original draft. YYZ: Data curation, Writing – original draft. YJZ: Formal analysis, Writing – original draft. MY: Formal analysis, Writing – original draft. LT: Writing – review & editing.
